# Infected Thyroglossal Duct Cyst Involving Submandibular Region: A Case Report

**DOI:** 10.1155/2011/978263

**Published:** 2011-08-24

**Authors:** Rahul A. Gandhi, Rahul Bhowate, Shirish Degweker, Arvind Bhake

**Affiliations:** ^1^Oral Diagnosis Medicine and Radiology, Sharad Pawar Dental College & Hospital, DMIMS University, Sawangi, Wardha 442004, India; ^2^General Pathology, Jawaharlal Nehru Medical College & Hospital, DMIMS University, Sawangi, Wardha 442004, India

## Abstract

Thyroglossal duct cyst presents most frequently in the midline of the neck, either at or just below the level of the hyoid bone. They generally manifest as painless neck swelling, and they move on protrusion of tongue and during swallowing. A case of thyroglossal cyst was reported in the left submandibular region in a 14-year-old girl, above the level of hyoid bone; ultrasound examination favored a cystic lesion which moved in a vertical fashion on swallowing whereas fine needle aspiration cytology report was suggestive of simple cystic lesion of thyroglossal cyst. No lymphoid or malignant cells were present. The cyst was excised completely by surgical procedure under general anesthesia. Histopathological analysis revealed thyroglossal cyst showing columnar and flattened epithelium of cyst with focal aggregate of chronic inflammatory cells supported by fibrocollagenous cyst wall. The clinical, ultrasound, and histopathological findings suggested that the lesion was an infected thyroglossal cyst. There was no evidence of recurrence 6 months after surgery.

## 1. Introduction

The primitive thyroid gland originates from the foramen cecum present in the floor of the pharyngeal gut on the 17th day of gestation. The gland then descends in front of the pharynx as a bilobed diverticulum which is initially patent. It reaches its final position in the neck by the 7th week of the gestation. During migration it is connected to the tongue by a narrow tubular structure—the thyroglossal duct. The duct usually disappears by the 10th week of gestation [1]. Persistence of any portion of this duct and secretion from the epithelium lining (likely to represent repeated local infection and inflammation) may give rise to the cystic lesion [2]. 

The thyroglossal cyst is the commonest congenital neck mass in children, accounting for 70% of congenital neck anomalies and second most benign neck mass, after lymphadenopathy [3]. On preoperative imaging workup of a patient with thyroglossal cyst, the following aspects need to be considered [4]. (a) Thyroid carcinoma can develop in a thyroglossal cyst, with an incidence of 1% in adults. (b) Normal thyroid tissue has to be identified by ultrasound in the anterior neck before surgery to prevent postoperative hypothyroidism. (c) The relationship of the TDC with the hyoid bone must be determined as it helps the surgeon to completely excise the lesion so reducing the chance of recurrence. Indications for excision may include cosmetic appearance, recurrent infections, sinus and fistula formation, and risk for malignant change [4–6]. The classic operation was described by Sistrunk (1920) and consists of excision of the thyroglossal cyst, the central portion of the hyoid bone and a core of tissue around the thyroglossal tract to open into the oral cavity at the foramen cecum [5]. 

Our objective is to present a rare case of infected thyroglossal duct cyst in left submandibular region.

## 2. Case Report

**Figure 1:**
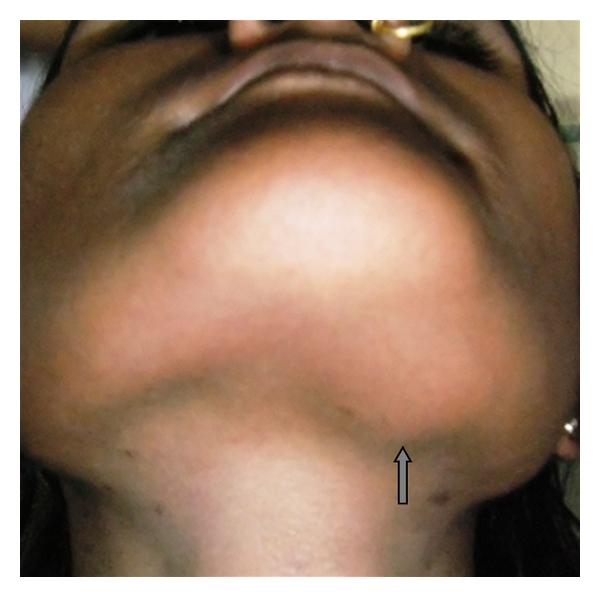
Swelling at the left submandibular region.

A 14-year-old girl from the low socioeconomic status, resident of rural area presented to Department of Oral-Medicine & Radiology, Sharad Pawar Dental College & Hospital, DMIMSU, Sawangi, Wardha, India, with the chief complaint of swelling on the left submandibular region with sore throat since 1 month. She has experienced pain in the same region while swallowing. The patient was afebrile with the temperature, pulse, blood pressure and respiration within normal limits. On examination, a 1.5 × 1.5 cm swelling was palpated under the middle one third of left Submandibular region; which was soft and slightly tender on palpation. The overlying skin was comparable with normal wheatish adjacent skin color. The swelling was mobile in vertical direction on swallowing (Figure 1). Intraoral examination revealed considerable blanching on both the buccal mucosa and soft palate with shrunken uvula. Vertical fibrous bands were more prominent on left buccal mucosa which leads to partial trismus with interincisal mouth opening of 28 mm. Clinical signs and positive corelation with betel nut habit led to mucosal diagnosis of oral submucous fibrosis.

**Figure 2:**
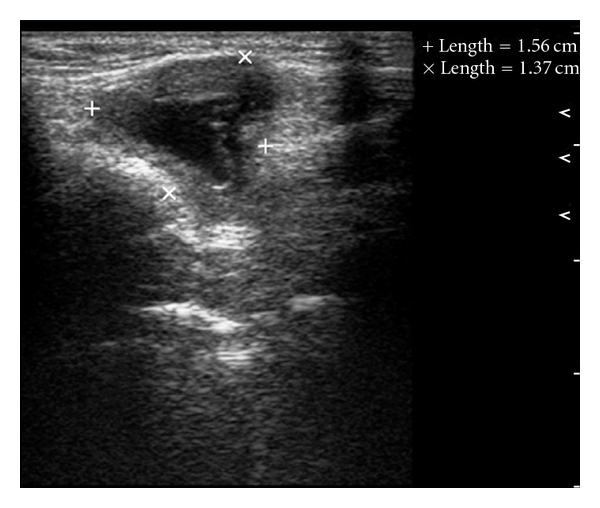
Longitudinal grey-scale ultrasound in the left submandibular region shows an anechoic TDC with thick wall.

**Figure 3:**
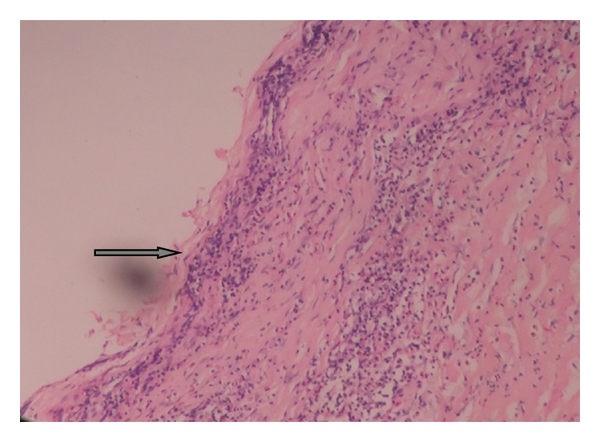
Photomicrograph (haematoxylin and eosin section; ×10 magnification) showing columnar and flattened epithelium of cyst with focal aggregate of chronic inflammatory cells supported by fibrocollagenous cyst wall.

Intraoral periapical radiographs with 34, 35, and 36 were within normal limits. Ultrasonographic examination of submandibular swelling showed well-defined cystic lesion with thick wall measuring 1.13 × 1.27 cm (Figure 2). Ultrasonic evaluation of thyroid gland showed that it was within normal position. Aspiration yielded clear viscous fluid. Fine needle aspiration cytology was suggestive of simple cystic lesion of thyroglossal cyst. No lymphoid or malignant cells were present. The preoperative chest radiograph, complete blood count, and urine analysis and blood chemistry revealed no abnormality. After assessment, provisional diagnosis of infected thyroglossal cyst was given. The cyst was surgically excised completely, under general anesthesia, and postoperative healing was uneventful. The specimen was sent to histopathologic examination. The histological haematoxylin and eosin section showed columnar and flattened epithelium lining of cyst with focal aggregates of chronic inflammatory cells supported by fibrocollagenous cyst wall (Figure 3). These histopathological findings confirmed the provisional diagnosis of infected thyroglossal cyst.

## 3. Discussion

The most common congenital anomaly related to thyroglossal duct is the thyroglossal duct cyst (TDC) located in the region of the hyoid bone. About 15 to 50% are at the level of hyoid bone, 20 to 25% are suprahyoid, and 25 to 65% are infrahyoid [12]. No gender predilection has been reported, and the age of the affected patient ranges from birth to 70 years; approximately 50% of patients present before the age of 20 years [7]. The cyst could lie anywhere within the thyroglossal tract, from the base of the tongue to the suprasternal region. About 90% of thyroglossal duct cysts lie at or very close to midline [7]. Remaining 10% lies on one side of the midline (mostly on left side) [7], which support the unusual location in our patient. Commonly in childhood it presents as a midline neck lump that is usually enlarging, painless, smooth, and cystic and if infected, pain can occur. There may be difficulty in breathing, especially if the lump becomes large. Generally, thyroglossal duct cyst moves with deglutition and on protrusion of the tongue. Infection can sometimes cause the transient appearance of a mass or the enlargement of the cyst, at times with periodic recurrences [6]. 

In the vast majority of cases, ultrasound supplemented by fine needle aspiration cytology is adequate for pretreatment assessment [8]. FNAC serves as complementary diagnostic method to histopathological examination [9, 10]. Thyroglossal duct cyst yields a clear yellow to whitish fluid. The smear is hypocellular and shows follicular cells, lymphocytes, and macrophages. According to the histopathological findings, the epithelial linings of the cyst may be pseudo stratified columnar, ciliated columnar, squamous, simple cuboidal, or transitional epithelium.

Mucous glands and thyroid follicles are commonly seen in the subjacent stroma. Secondary inflammatory changes are usually found. The cytological findings of a thyroglossal cyst are similar to those of a thyroid cyst. However, squamous epithelial cells are usually not noticed as components of a thyroid cyst. This may be the only significant cytomorphologic difference between a thyroglossal duct cyst and a thyroid cyst [11]. Detailed histological examination is essential not only to establish the diagnosis of TDC but also to rule out carcinoma. No ectopic thyroid was found in our patient.

The typical sonographic description of a thyroglossal duct cyst has been that of an anechoic, well-circumscribed cyst [8]. The pseudo solid appearance of cystic lesions may be due to proteinaceous content of the fluid, thought to be secreted by the epithelial lining of the cyst. Solid component in the cystic wall is required in sonography to consider it as carcinoma. A “Sistrunk” procedure is the recommended procedure of choice [5]. The recurrence rate following this procedure is 5% [5]. In differential diagnosis, dermoid cyst, infected lymph node, lipoma, sebaceous cyst, branchial cyst, laryngocele, and cystic hygroma should be considered [10]. Lymph nodes are often multiple and hypoechoic and show the presence of echogenic hilus. On aspiration, there is cheesy secretion from dermoid cyst, purulent secretion from infected lymph node, and air from laryngocele and branchial cyst aspirated materials shows fat globules and cholesterol crystals [11–13]. Lipoma has slippery edges, and sebaceous cyst has doughy feel. In cystic hygroma transillumination is brilliantly positive as compared to TDC. 

In the present case, the history of sore throat and pain on swallowing was suggestive of infected cyst. The cyst moved during swallowing and on protrusion of tongue which directed the diagnosis towards thyroglossal duct cyst. Moreover, the histopathological report has established confirmed diagnosis with its characteristic features.
